# Supplementary material for: Pediatric emergency care in a low-income country: Characteristics and outcomes of presentations to a tertiary-care emergency department in Mozambique
Source: PLoS One. 2020 Nov 4;15(11):e0241209. doi: 10.1371/journal.pone.0241209 (PMC7641453; doi:10.1371/journal.pone.0241209)
Supplement: S1 Table — The valid cases (total of data available) have been reported with the number of hospitalized patients. Univariable analysis results are reported in the table. (DOCX) [file pone.0241209.s001.docx]

|  |  | Total of Data available  N 28,844 | **Hospitalization**  **N: 4,997** | **Univariate Analysis**  **OR (95% CI)** | **p – value** | **Mortality in the PED**  **N: 396** | **Univariate Analysis**  **OR (95% CI)** | **p – value** | **Early death**  **(≤ 4 h)**  **N: 247** | **Univariate Analysis**  **OR (95% CI)** | **p –value** |
| --- | --- | --- | --- | --- | --- | --- | --- | --- | --- | --- | --- |
| SEX | **Male** Vs | 14,448 | 2,983 | 0.98 (0.92 - 1.05) | 0.59 | 182 | 1.02 (0.81 - 1.28) | 0.89 | 90 | 0.74 (0.47 – 1.18) | 0.206 |
|  | Female | 10,114 |  |  |  |  |  |  | 71 |  |  |
| AGE |  |  | 4,997 |  |  | 396 |  |  | 247 |  |  |
|  | 0 – 28 days | 677 | 201 | 2.81 (2.34 - 3.37) | **< 0.001** | 109 | 25.93(18.93 - 35.51) | **< 0.001** | 97 | 8.31 (3.90 – 17.68) | **< 0.001** |
|  | 29 d – 1 year | 5,634 | 1,422 | 1.77 (1.63 - 1.93) | **< 0.001** | 96 | 2.04 (1.5 - 2.77) | **< 0.001** | 57 | 1.50 (0.81 – 2.77) | 0.194 |
|  | 1 – 5 years | 10,845 | 2,132 | 1.27 (1.18 - 1.37) | **< 0.001** | 118 | 1.2 (0.89 - 1.61) | 0.23 | 57 | 0.96 (0.54 – 1.72) | **0.892** |
|  | 5 – 15 years ‡ | 7,688 | 1,242 |  |  | 73 |  |  | 36 |  |  |
| RESIDENCY | Urban Vs | 22,948 |  |  |  |  |  |  | 230 |  |  |
|  | **Extrasofala** | 1,887 | 818 | 3.52 (3.19 - 3.88) | **< 0.001** | 43 | 2.19 (1.58 - 3.02) | **< 0.001** | 17 | 0.35 (0.18 – 0.67) | **0.002** |
| MODALITY of PRESENTATION | Self-presentations Vs | 14,477 |  |  |  |  |  |  | 178 |  |  |
|  | **Health Care Provider referral** | 10,360 | 3,380 | 3.87 (3.62 - 4.13) | **< 0.001** | 165 | 1.32 (1.08 - 1.62) | **0.01** | 69 | 0.29 (0.19 – 0.45) | **< 0.001** |
| PRESENTING COMPLAINT | **Medical** Vs | 9,522 | 3,607 | 5.75 (5.17 - 6.39) | **< 0.001** | 48 | 1.32 (0.82 - 2.14) | 0.25 | 19 | 0.76 (0.29 – 2.00) | 0.585 |
|  | Injury | 4,682 |  |  |  |  |  |  | 12 |  |  |

**S1 Table.** **Determinants of hospitalization, mortality in the PED, and early death.** The valid cases (total of data available) have been reported with the number of hospitalized patients. Univariate analysis results are reported in the table.
