# Supplementary material for: Pediatric emergency care in a low-income country: Characteristics and outcomes of presentations to a tertiary-care emergency department in Mozambique
Source: PLoS One. 2020 Nov 4;15(11):e0241209. doi: 10.1371/journal.pone.0241209 (PMC7641453; doi:10.1371/journal.pone.0241209)
Supplement: S2 Table — Absolute numbers and percentages of hospitalized patients have been reported. Univariable Analysis (OR, 95% Confidence Intervals (CI), and p-values, adjusted for gender, age, residency, and modality of presentation) are represented in the table. (DOCX) [file pone.0241209.s002.docx]

**S2 Table.** **Association between presenting complaints and hospitalization**. Absolute numbers and percentages of hospitalized patients have been reported. Univariable Analysis (OR, 95% Confidence Intervals (CI), and p-values, adjusted for gender, age, residency, and modality of presentation) are represented in the table.

|  |  |  | **N^** | **Hospitalization** | **Univariate Analysis** | **p - value** |
| --- | --- | --- | --- | --- | --- | --- |
| **PRESENTING REASON** | | |  | **(n)^^** | **OR (95% CI)** |  |
|  |  | **Medical** | 9,522 | 3,607 |  |  |
|  |  | Fever ‡ | 2,540 | 754 (21%) |  |  |
|  |  | Respiratory | 2,789 | 873 (24%) | 1.08 (0.96 - 1.21) | 0.194 |
|  |  | Neurological | 752 | 465 (13%) | 3.81 (3.22 - 4.52) | **<0.001** |
|  |  | Gastrointestinal | 1,355 | 461 (13%) | 1.22 (1.06 - 1.41) | **0.005** |
|  |  | Cardiovascolar | 77 | 64 (2%) | 11.67 (6.39- 21.31) | **<0.001** |
|  |  | Muskoloskeletal | 225 | 81 (2%) | 1.11 (0.84 - 1.46) | 0.462 |
|  |  | Constitutional* | 794 | 638 (18%) | 9.63 (7.93 - 11.70) | **<0.001** |
|  |  | Sense Organs** | 936 | 242 (7%) | 0.82 (0.70 - 0.98) | **0.027** |
|  |  | Others*** | 54 | 29 (1%) | 2.75 (1.60 - 4.73) | **<0.001** |
|  |  |  |  |  |  |  |
|  |  | **Injury** | 4,682 | 450 |  |  |
|  |  | Drowning | 15 | 1 (0%) | 1.12 (0.15;8.56) | 0.91 |
|  |  | Road Accident | 457 | 84 (19%) | 3.53 (2.66 - 4.68) | **<0.001** |
|  |  | Fall ‡ | 2,949 | 175 (39%) |  |  |
|  |  | Burn | 237 | 109 (24%) | 13.34 (9.91 - 17.97) | **<0.001** |
|  |  | Wound | 401 | 36 (8%) | 1.55 (1.06 - 2.25) | **0.022** |
|  |  | Violence | 143 | 3 (1%) | 0.34 (0.11 - 1.06) | 0.063 |
|  |  | Ingestion/Inhalation | 480 | 42 (9%) | 1.50 (1.06 - 2.14) | **0.023** |

*lethargy, weakness, loss of appetite, fatigue, etc. ** sense organs are defined as the body organs by which humans can see, smell, hear, taste and touch or feel. This category includes medical complaints to the eyes, ears, nose, throat, and the skin. ***Psychiatric and genitourinary diseases

‡ Reference Category
