# Supplementary material for: Pediatric emergency care in a low-income country: Characteristics and outcomes of presentations to a tertiary-care emergency department in Mozambique
Source: PLoS One. 2020 Nov 4;15(11):e0241209. doi: 10.1371/journal.pone.0241209 (PMC7641453; doi:10.1371/journal.pone.0241209)
Supplement: Attachment — Submitted filename: Response to reviewers 2 DEF.docx [file pone.0241209.s004.docx]

Dear Editor in Chief, Prof Itamar Ashkenazi,

Thank you for your thorough and timely review of our revised manuscript **"Pediatric emergency care in a low-income country: characteristics and outcomes of presentations to a tertiary-care emergency department in Mozambique".**

We have responded point by point to all the comments made by the reviewers in our rebuttal letter below. As per instructions, we also uploaded a marked-up copy as a separate file labeled 'Revised Manuscript with Track Changes'. We uploaded the clean version of our revised paper without tracked changes as a separate file labelled 'Manuscript'.

We are looking forward to hearing from you.

Sincerely yours.

Best regards,

Dr. Valentina Brugnolaro

(corresponding author)

**Reviewers' comments:
Review Comments to the Author**

Reviewer #2: The authors have been very responsive to reviewers’ comments. They have performed additional analyses and have been very thorough in assessing the limitations of their study. I applaud the authors for their responsiveness. I think the article is much strengthened at this point and I feel this is a good beginning to further elucidate what type of patients and outcomes occur in a single PED in Mozambique. My comments are extremely minor at this point.

***Author’s reply****: We thank the reviewer for the supportive comment.*

-If the authors had a “two-month data abstraction overlap between the abstractors helped in ensuring consistency in data abstraction and coding”, should there be a kappa calculated to determine the inter-rater agreement?

***Author’s reply:*** *We thank the reviewer for this comment.* *The two-month data abstraction overlap was used for training purposes of the second data abstractor, and for clarification of possible doubts. There was no double entry of all data, so unfortunately, we cannot determine the inter-rater agreement. To clarify this point, we added the following wording to the sentence "...consistency in data abstraction and coding****, by training of the second data abstractor. No formal double entry of data by the two abstractors occurred during this time****.”*

-What was the p-value cut off the authors used to determine “significant” in the univariate model to then include in the multivariable model? Could be added around line 142.

***Author’s reply:*** *The p-value cut-off we used to include significant variables at the univariate model into the multivariable model was 0.05. We have added this information to the manuscript, as suggested by the Reviewer.*

-Line 166: “already been” could be reworded. Consider “42% had been assessed by a health care provider…”

***Author’s reply:*** *We have revised the sentence accordingly.*

-Consider presenting the variables in Tables 4 and 6 on different rows to make it clearer which variable is being reported on some lines. The authors appear to use bold text to do this, but I think splitting variables like “Health care provider referral” and “Self-presentation” as separate rows. This will help with the multivariate reporting to as it will emphasize what the referent is.

***Author’s reply:*** *We have revised tables 4 and 6 according to the reviewer suggestions.*

Reviewer #3: The author has well answered point by point to all the comments. Since, the manuscript has been substantially improved and I don't have any additional comments to the author.

***Author’s reply****: We thank the reviewer for the supportive comment.*

Reviewer #4: General thoughts:
- This is a retrospective chart review with a primary objective of determining systems-level interventions and design features for an effective pediatric emergency medicine system in a low-income (Mozambique) country. The authors responses were thorough and appreciated.

***Author’s reply****: We thank the reviewer for the supportive comment.*

Abstract:
- A major finding in this paper is the lack of a “paper trial” or system for data records (i.e 43% of presenting complaint data is missing, the lack of multiple years of data to review). This finding shouldn’t simply be relegated to a limitation of the study. It should be a finding that is embraced as a major barrier to improving the care of children presenting for PED care in this country. I would love to see this finding reported on the abstract.

***Author’s reply****: As suggested, we have now included this finding in the Results section of the abstract and commented on it in the conclusion section of the abstract.*

Materials and Methods:

- This revised section flows much better. The inclusion of information about the ward set-up and layout is of the PED and the Pediatric Healthcare system is much appreciated and flows well.

***Author’s reply****: We thank the reviewer for the supportive comment.*

- There seem to be two captions for Figure 1 (place clarify where the location of this figure is meant to be placed. The title for Figure 1 seems like it could be more like a result was reported. I would include the keyword: registries (i.e. something to the effect of Figure 1: Study register characteristics.

***Author’s reply****: The Reviewer is right, in the marked-up copy of the manuscript the caption appears twice. We realized that the paragraph referring to Figure 1 is duplicated in this version of the manuscript, while the clean version is correct and the Figure caption appears only once. As suggested, we have reworded Figure 1 caption as follows:* ***"******Characteristics of hospital registries from which study data were collected"****.*

- We are unable to see Figure 1, and hence cannot comment on it.

***Author’s reply:*** *We are sorry that the Reviewer was unable to see figure 1. We followed the instructions for the upload of figures and could see it in the pdf of the manuscript for download. We hope there are no technical issues preventing the inclusion on the figure in the revised version of the manuscript.*

- Please include in the manuscript your rationale for why ICD9/10 codes are not used.

***Author’s reply****: As recommended by the Reviewer we have included the following sentence in the revised version of the manuscript, at the end of the section on "Sources of data and data collection procedures": "****Unfortunately the HBC did not have the facilities and resources (i.e., trained staff, information technology infrastructure) to code diagnosis according to the ICD 9/10 codes. Data on diagnosis were reported as per local documentation practices****".*

- The new sensitivity analysis is a nice touch.

***Author’s reply****: We thank the reviewer for the positive comment.*

o Typo in point 3. Should read “outcomes”, not “otcomes”

***Author’s reply****: Thank you for noticing it, we have corrected the typo accordingly (line 154).*

Results:
- Please check spacing of the first row “mortality” in Table 3. Please make sure all the chart lines up (in admission section)

***Author’s reply****: We thank the reviewer for noticing this, we have corrected the spacing accordingly (Table 3)*

- Data is presented well an in an organized fashion. Tables are excellent.

***Author’s reply****: We thank the reviewer for the positive comment.*

- Limitations is presented in the Results section (page 19). Confusing to same it the same name as a section usually found in the discussion. Would rename this subtitle according to the primary finding regarding limitations of data sources (which is not a limitation of the methods and study design per se, but a separate stand-alone finding).

***Author’s reply****: We thank the Reviewer for the suggestion. The limitation section was adjusted and reported just after the Results and before the Discussion following a previous recommendation from another Reviewer. We are aware some Journals prefer for the Limitations section to appear before the discussion rather than at the end of the discussion. We are happy to follow the Editors' recommendations as to where the Limitations section should be placed in the manuscript.*

Discussion:

- Rewritten discussion and focus on recommendations from data is much better understood. The relation to WHO and UNICEF guidelines is a good direction to take.

***Author’s reply****: We thank the reviewer for the supportive comment.*
